# Small-Pox.—Vaccination

**Published:** 1891-02

**Authors:** W. M. Yandell

**Affiliations:** Health Physician, El Paso, Texas


For Daniel's Texas Medical Journal.

### SMALLPOX—VACCINATION.

BY W. M. YANDELL, M. D., HEALTH PHYSICIAN, EL PASO, TEXAS.

The Brenham correspondent of the *Galveston News*, reports Dr. Rutherford, then State Health Officer, holding the opinion that vaccination is not a safeguard against smallpox. Whether or not Dr. Rutherford is correctly reported is not to the point. The bald fact remains that the then State Health Officer, supposed from his position to be well up in preventive medicine, has been reported in the leading newspaper in the State as holding such opinion; the harm has been done and a dozen corrections through the same paper will not undo it. El Paso, the largest city on the Mexican border, was the only one on the border not included in Gov. Ross' ill-advised, ill-timed and useless proclamation, which did the State incalculable damage, and the effect of vaccination both in preventing and modifying smallpox in this city is worth recording.

The first case of smallpox in the city for years, was reported December 14, 1889, and from that date until June, 1890, date of the last case during the year, there were thirty cases in all. Of these, fourteen were in persons never vaccinated at all, or never successfully vaccinated, and singularly enough, not one was discrete, but all of the confluent type. Seven of these fourteen died and seven recovered.

There were ten cases in persons vaccinated successfully but once, and one case in a woman who claimed to have been successfully vaccinated twice. All of these were varioloid, all recovered; most of them were not sick enough to go to bed after the eruption appeared, and, in fact, two were, when reported, walking about the streets not knowing what they had.

The other five cases were in Mexicans who crossed over to Mexico and I have no record of them.

Again: A woman and her two children went with her husband, who had confluent smallpox, to the smallpox hospital. She and the younger child had never before been vaccinated but were successfully vaccinated after they had contracted the disease (as was shown by the fact that the primary fever in both made its appearance in seven days after the vaccination); both were of the confluent type, and both recovered, although she was seven months gone in pregnancy, and of course miscarried. That the successful vaccination modified the disease in each, I cannot of course prove, but my opinion is, that it did, though so late. The remarkable point remains to be stated: The other child had been attending the city public schools where the compulsory vaccination rule is strictly enforced, and had been successfully vaccinated some three months before. *He slept, ate and lived for over a month with the three others in all stages of the disease, and escaped.*

Another woman, with the most thoroughly marked case of varioloid that I had, carried three children with her to the smallpox hospital. They ate and slept in the same room with her, yet none of them contracted the disease, being protected by successful vaccination.

The hospital keeper waited on seventeen cases that were moved to the hospital. He was protected by a successful vaccination when a child and a successful re-vaccination two months before our first case. His two children, successfully vaccinated at the time of his re-vaccination, remained in the hospital, and none of them contracted the disease. His wife had smallpox when a child.

I attended eighteen of the cases, and saw others. I am a protected by a successful vaccination in infancy and successful re-vaccination at eighteen, and another when I was treating the second case, December, 1889. Of course I did not have the disease.

Few Mexican children, comparatively, are vaccinated in the town of Jaurez (formerly Paso del Norte), across the river from, and about the same size of this city. Fifty deaths from smallpox occurred there from October, 1889, to June, 1890, while during the same period in El Paso there were seven deaths from that disease. No record of the number of cases in Jaurez could be obtained as none is kept.

Under orders of the Board of Health of this city, I make a house to house inspection of the Mexican quarters every fall, and vaccinate, with the assistance of a policeman if necessary, as it sometimes is, all children not protected by successful vaccination or by previous attacks of smallpox. The grown people have usually had smallpox, only a few being protected by vaccination. Numbers of the children who have come from Mexico have had the disease. I vaccinated over 250 children for the city the fall of 1889, and have vaccinated over 200 this season. With the school children vaccinated, with the children of the well-to-do vaccinated at their own expense, with the children of the poor vaccinated by the city, and with nearly all the adults of the town protected by vaccination (and previous smallpox among the Mexicans), we have not the material for a smallpox epidemic, consequently have escaped the disease, although it has prevailed in Southern New Mexico, within twenty miles or less of our city ever since it disappeared here.

When a case is reported to me it is allowed to remain at home if the house is sufficiently isolated, and the family will pay for guards day and night, until the termination of the case. No one but the medical attendant is allowed to enter and leave the house. On the termination of the case all bedding and clothing used by the patient are burned, and everything else about the house disinfected with a 1-500 bi-chloride of mercury solution, after which the rooms are closed tightly, and fumigated with roll sulphur, about eight or ten pounds to each 1000 cubic feet of space. My reliance is on the bi-chloride.

Except in the case of a boy who had such a light case of varioloid that no doctor was called, and whose sister contracted the disease from him through carelessness of parents, no one else contracted the disease from any of the cases embraced in this report (except the woman and child who went to the hospital with the husband).

All persons not in isolated houses and able to pay for guards, are sent to the smallpox hospital.

Of the thirty cases there were sixteen whites, six negroes, six Mexicans, and two Spaniards, who from their carelessness should be classed with the Mexicans. The population of El Paso is, whites, over 8000; Mexicans, over 2000; negroes, about 300.

Three cases were in non-residents who contracted the disease in Mexico, and a majority of the others were directly traceable to that country. Ten of the number were directly traceable to gambling dens where Mexicans most do congregate.

Only a case or two was among the well-to-do population. Only one, varioloid, was in a school child, the one reported above as contracting it from her brother through carelessness of her parents. The maximum penalty for failing to report any contagious disease, is a fine of $100 and thirty days in jail. The careless father above mentioned was made to pay a fine for his failure to report his boy although the boy was well and on the street when I discovered him.

It is a mistake to suppose that a person who has been vaccinated a number of times unsuccessfully is not liable to smallpox. I lost a woman with confluent smallpox last year, who had been vaccinated unsuccessfully a number of times.

The record of the London smallpox hospital is fifty per cent. of deaths in confluent smallpox. I have not broken the record.

The ridiculous smallpox scare over the State, which received a fearful impetus from the Governor's proclamation, would be laughable if it were not so serious. If it is proposed to quarantine against smallpox no small army will be necessary on the Mexican border, as it prevails constantly somewhere in that country, and the Rio Grande is fordable anywhere on the northern border for several hundred miles about six months every year. Something may be done by intelligent inspection in the border towns, if the Mexicans can be induced to co-operate, much may be done; if vaccination is properly enforced all over Texas nothing more is necessary.

I have nothing to offer as to treatment, except to say that the following has proved remarkably efficacious in preventing the intolerable itching (and I believe pitting) of the disease:

℞ Acid salicylic, ʒ iijss.

Starch, ℥ jr.

Glycerine, ℥ jx.

M. Sig. Apply to hands, face, etc., with camel's hair brush as needed.

A milder form suitable for small children is:

℞ Salol, Starch, } *Equal parts.*

Glycerine q. s. to make a paste.

Apply as above.

The people should be taught that more than one successful vaccination is, as a rule, required to furnish perfect immunity from smallpox. In this city adults or children past puberty, with only one successful vaccination are the exception. Yet of the thousands who have had more than one successful vaccination only one was attacked last year, and that was a mild case of varioloid. Personally, I would not toss coppers for choice of immunity furnished by three successful vaccinations or by a previous attack of smallpox.

